# Transcriptomics reveals a cross-modulatory effect between riboflavin and iron and outlines responses to riboflavin biosynthesis and uptake in *Vibrio cholerae*

**DOI:** 10.1038/s41598-018-21302-3

**Published:** 2018-02-16

**Authors:** Ignacio Sepúlveda-Cisternas, Luis Lozano Aguirre, Andrés Fuentes Flores, Ignacio Vásquez Solis de Ovando, Víctor Antonio García-Angulo

**Affiliations:** 1 Programa de Microbiología y Micología, Instituto de Ciencias Biomédicas, Universidad de Chile, Santiago, Chile; 2 Escuela de Biotecnología, Universidad Mayor, Campus Huechuraba, Santiago, Chile; 3 Centro de Ciencias Genómicas, Universidad Nacional Autónoma de México, campus Chamilpa Cuernavaca, Morelos, Mexico

## Abstract

*Vibrio cholerae*, a pandemic diarrheagenic bacterium, is able to synthesize the essential vitamin riboflavin through the riboflavin biosynthetic pathway (RBP) and also to internalize it through the RibN importer. In bacteria, the way riboflavin biosynthesis and uptake functions correlate is unclear. To gain insights into the role of the riboflavin provision pathways in the physiology of *V. cholerae*, we analyzed the transcriptomics response to extracellular riboflavin and to deletions of *ribD* (RBP-deficient strain) or *ribN*. Many riboflavin-responsive genes were previously reported to belong to the iron regulon, including various iron uptake genes. Real time PCR analysis confirmed this effect and further documented that reciprocally, iron regulates RBP and *ribN* genes in a riboflavin-dependent way. A subset of genes were responding to both *ribD* and *ribN* deletions. However, in the subset of genes specifically affected in the ∆*ribD* strain, the functional terms *protein folding* and *oxidation reduction process* were enriched, as determined by a Gene Ontology analysis. In the gene subset specifically affected in the ∆*ribN* strain, the *cytochrome complex assembly* functional term was enriched. Results suggest that iron and riboflavin interrelate to regulate its respective provision genes and that both common and specific effects of biosynthesized and internalized riboflavin exist.

## Introduction

Redox reactions, consisting of electron transfers from an oxidizing molecule to a reducing one, lie at the core of many central physiological processes. These include oxidative phosphorylation, cell signaling, photosynthesis, DNA repair, carbohydrates metabolism, oxygen storage, photosensitization and protein folding among many other^1–3^. In order to complete these reactions, enzymes usually require redox cofactor molecules which include nicotinamide-derived molecules, iron-sulfur clusters, thiamin, deazaflavin and transition metals like cooper, manganese, cobalt and zinc^3–8^. However, iron is by far the most widespread metal redox cofactor, while molecules derived from riboflavin (also named vitamin B2), such as flavin mononucletoide (FMN) and flavin adenine dinucleotide (FAD) constitute the main organic electron transfer cofactors, with an importance similar to that of iron^6^. Genes encoding flavoproteins may comprise up to 3.5% of the genome of a species^9^. Flavins are probably the most versatile cofactors, being able to catalize one- and two-electron transfers, which allows their participation in electron bifurcation reactions^10^. These molecules may also catalize non-redox reactions and are increasingly recognized as covalent catalysts, acting in the formation of flavin-substrate adduct intermediates^9,11^.

There is evidence that flavins may act as signaling molecules in bacteria. Riboflavin and its breakage derivative lumichrome are able to mimic N-acyl homoserine lactone for activation of quorum sensing pathways in *Pseudomonas aeruoginosa* and riboflavin is a chemoattractant to *S. oneidensis*^12,13^. Riboflavin may as well be secreted by some bacteria to be used as electron shuttle to reduce Fe^+3^ into its more soluble Fe^+2^ form and to complete the extracellular respiratory chain^14–17^. In addition, this vitamin frequently represents a metabolic currency during bacteria-host or intermicrobial trade interactions^18,19^.

Most bacteria are able to biosynthesize riboflavin through the riboflavin biosynthetic pathway (RBP). This pathway starts with guanosine triphosphate (GTP) and ribulose-5-phosphate to synthesize riboflavin using the RibA (GTP cyclohydrolase II), RibD (pyrimidine deaminase/reductase), RibH (lumazine synthase), RibB (3,4-dihydroxybutanone phosphate synthase) and RibE (riboflavin synthase) enzymes^20,21^. The nomenclature of RBP enzymes varies among bacterial species and *Escherichia coli* names^22^ are thoroughly used here. In bacterial genomes, RBP genes could form an operon or be positioned in different loci. In various species, some RBP genes are duplicated or multiplicated^23^. In some cases, duplicated RBP gene orthologs appear to implement modularity to riboflavin production, where the RBP uses subsets of genes to provide riboflavin for specific purposes, such as secretion or interactions with the host^24,25^. Bacteria may also use importer proteins to internalize riboflavin from the surroundings. Although many bacterial species rely exclusively on riboflavin uptake, many others possess both riboflavin biosynthesis and uptake. It is hypothesized that this overlay allows bacteria to take advantage of changing environments, turning on riboflavin uptake and stopping biosynthesis in nutrient rich niches, while granting autonomy when facing stringent conditions. It is also posible that riboflavin importers procure flavins for specific functions in riboflavin-prototrophic species^23,26–28^.

*Vibrio cholerae* are Gram negative proteobacteria responsible for cholera, a pandemic disease affecting mainly developing countries, characterized by acute, life-threatening diarrhea^29^. Global cholera burden has recently been estimated in around 2.8 million cases with 95,000 deaths per year^30^. Most *V. cholerae* strains are innocuous indigenous members of estuarine and seawater microbiota, with a few strains from serotypes O1 and O139 causing almost all of cholera cases^29–32^. In these bacteria, development of virulence is not only associated with the acquisition of virulence factors but also of specific alleles of virulence adaptive polymorphisms rotating in environmental species, which confer selective advantages like host colonization properties^32^. Importantly, environmental water conditions such as temperature, salinity, pH and sunlight exposure have a major impact in the development of cholera epidemics and thus outbreaks are expected to increase due to global warming^31,33^.

Cholera is mostly a waterborne disease, and after human consumption, *V. cholerae* expresses several virulence factors. Cholera toxin is the main inducer of diarrhea. This toxin translocates into host epitelial cells to promote constitutive activation of the adenylate cyclase, causing an increase in Cl^−^ and water efflux. Initial adhesion to host intestine is promoted by the toxin coregulated pilus. In addition, other *V. cholerae* virulence factors such as the flagellum, the HapA metalloprotease, Zot and RTX toxins and different iron acquisition systems are also expressed in order to favor host colonization^33,34^. In the environment, *Vibrio cholerae* is primarily found associated to abiotic surfaces and to chitin carpaces of acquatic organisms as microcolonies or biofilms, but also as planktonic cells. Biofilm formation is required during the host pathogenic phase and biofilm structures are detected in faeces from infected humans^34^. In addition, these bacteria are able to enter metabolically dormant viable but non culturable and persister states in response to harsh environmental conditions, which may allow bacteria to face physical and nutritional changes in niches or to survive in atypical environments such as fomites^35,36^. Thus, this bacterium has a complex life cycle and likely, both *V. cholerae* riboflavin requirements and availability are highly variable among the different environments and physiological states in which it may be found. Although there is no estimation of the number of flavin-requiring proteins in *V. cholerae*, a structural genomics approach calculated the proportion of genes coding for flavoenzymes in more than 1% in the related species *Vibrio fischeri*^9^. *V. cholerae* encodes a full RBP organized into a large operon and two monocistronic units. Together with genes not directly involved in riboflavin biosynthesis, the RBP operon contains *ribD*, *ribE*, *ribH* and a gene belonging to a family of hybrid *ribBA* genes common in proteobacteria. In addition, RibA and RibB monocystronic homologs are encoded in the genome of *V. cholerae*^37,38^. The *ribB* gene conserves a putative FMN riboswitch, which is a regulatory element forming alternative structures in the 5´ untranslated region of the messenger RNA to control expression depending on FMN binding status. The RBP is dispensable when *V. cholerae* grows in rich medium^39^, as this species also has a RibN riboflavin importer^40^. Unlike some orthologs in other proteobacteria, *ribN* in *V. cholerae* lacks a FMN riboswitch. We recently reported that when growing in the presence of extracellular riboflavin in standard minimal media, the expression of the monocistronic *ribB* gene is diminished while expression of the rest of the RBP genes and of *ribN* is not affected^38^.

In spite of the ubiquitous importance of riboflavin in bacterial physiology, no high throughput approach has been applied to study the response elicited by any bacterial species to this metabolite. Given the complex ecophysiological features of *V. cholerae*, this organism may comprise a suited model to study the way riboflavin biosynthesis and transport interplay to accomplish bacterial riboflavin needs. This study analized the transcriptomics response to extracellular riboflavin and compared the effects of the elimination of endogenous biosynthesis or uptake through the RibN importer. This allowed the identification of a set of genes responding to exogenous riboflavin, as well as to outline specific effects of synthesized or internalized riboflavin.

## Materials and Methods

### Strains and growth conditions

*V. cholerae* N16961 strain and its ∆*ribD* and ∆*ribN* derivatives were grown overnight in LB plates at 37 °C. 5 ml of LB broth were inoculated with a colony of the plate cultures and incubated at 37 °C in an orbital shaker at 150 rpm until they reached an OD_600nm_ of 1.0. Next, cultures were centrifuged and pellet washed twice with T minimal medium^41^ and resuspended in 1 ml of fresh T. 10 ml of plain T medium or T + 2 µM riboflavin were inoculated with 10 µl of the resuspensions and incubated at 37 °C and 180 rpm until an OD_600nm_ of 0.8. 1 ml of each culture was centrifuged and subjected to RNA extraction. When indicated, iron was omitted in T media and 3 ml of cultures at OD_600nm_ = 0.3 were harvested for RNA extraction. This growth protocol was performed three times independently for each condition and was similar for RNA subjected to transcriptomics and Real Time PCR (RT-PCR).

### RNA extractions, retrotranscription, RNAseq and RT-PCR

RNA extraction was performed with the Thermo Scientific Genejet RNA purification kit according to manufacturer’s instructions. RNA extracts were digested with Turbo DNA-free DNAase at 37 °C for 1 hour. For RNAseq, rRNA was removed using the Ribo-Zero removal kit and cDNA libraries were constructed using the TruSeq mRNA stranded kit, according to manufacturer’s instructions. Next, RNA was sequenced using the Illumina HySeq platform to produce 100 bp paired-end reads, with ~40 million reads per sample. Sequencing raw data files, processed sequence data files and metadata information was deposited at the Gene Expression Omnibus database from NCBI (GSE107538). rRNA removal, cDNA libraries generation and RNAseq were performed at Genoma Mayor (Santiago, Chile).

For RT-PCR analysis, the AffinityScript QPCR cDNA Synthesis kit (Agilent Technologies) was used for cDNA synthesis according to manufacturer’s instructions. As a negative control, a reaction with no reverse transcriptase was included for each sample in each run. RT-PCR was performed using the Brilliant II SYBR Green QPCR Master Mix kit in a One-Step Applen Biosystems (Life Technologies) thermocycler. Relative expression in the indicated conditions was determined through the ∆∆Ct method as developed before^42^. The 16 s ribosomal RNA gene was used for normalization. For the assessment of the relative expression by RT-PCR of *ribB*, *ribN*, *ribD* and *gyrB*, the sets of primers used were ribB Fw/ribB Rv, ribN Fw/ribN Rv, ribD Fw/ribD Rv and gyrB Fw/gyrB Rv^38^, respectively. Other RT-PCR primers are as follows: for *tonB1*, tonB1 Fw (5′- GGTGTTTGCCATGCCTGCTGG-3′)/tonB1 Rv (5′-GCGGCTTCACCTTCGGCTTAG-3′); for *sodA*, sodA Fw (5′-GCCAAGCGATATTCATCCAAGG-3′)/sodA Rv (5′-GCTCAGTGGCCTATCTTCATGC-3′).

### RNAseq data analysis

Quality control visualization and analysis (adapter and quality trimming) was performed using FastQC version 0.11.2 (http://www.bioinformatics.bbsrc.ac.uk/projects/fastqc/) and Trim_galore version 0.4.1 (http://www.bioinformatics.babraham.ac.uk/projects/trim_galore/), respectively. Reads were mapped to the genome of *Vibrio cholerae* 01 biovar El Tor str. N16961 (RefSeq, NCBI) using Bowtie2 version 2.1.0^43^. In all of the samples the alignment percentage of reads was above 98%. Differential expression analysis between samples was performed with the Bioconductor package edgeR version 3.18.1^44^ using negative binomial model and exact test based on quantile-adjusted conditional maximum likelihood method (qCML). Genes with a statistically significant change in expression (P < 0.05) were selected for further analysis. Analyses of enrichment of Gene Ontology (GO) terms of biological processes in the indicated subsets of genes were performed on the online platform of the Gene Ontology Consortium (www.geneontology.org), and statistically significant (P < 0.05) functional terms were retrieved.

## Results

### Overview of the experiment

**Figure 1:**
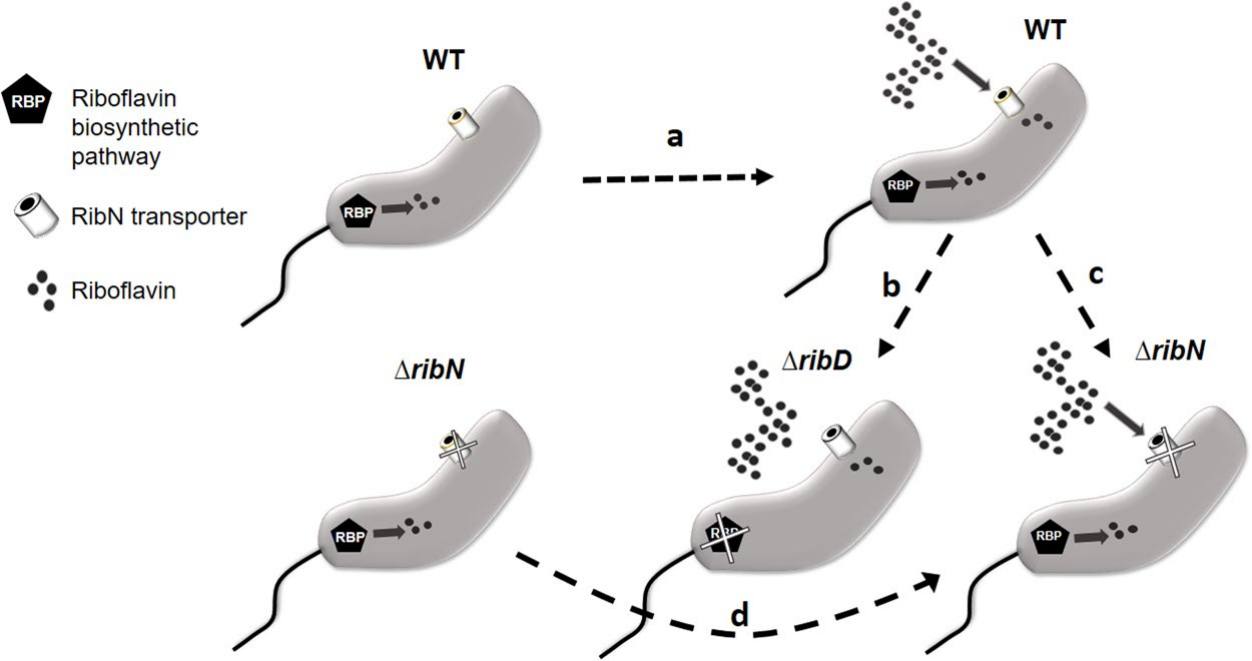
Schematic representation of the *V. cholerae* strains and conditions compared in the transcriptomics analysis. *V. cholerae* WT and its derivative mutant strains were grown in minimal T medium with or without riboflavin as indicated. Four transcriptomics comparisons were performed. In order to identify genes whose expression is regulated by riboflavin, transcriptomes of WT in T versus WT in T plus riboflavin were compared (comparison (**a**)). Comparison of WT versus Δ*ribD* (**b**) allowed to identify genes affected by the lack of riboflavin biosynthesis. Comparison (**c**) identified genes affected by the lack of riboflavin transport through RibN. Finally, comparison of the Δ*ribN* strain with and without riboflavin pinpointed genes affected by riboflavin independently of its uptake through RibN.

In *V. cholerae*, exogenous riboflavin downregulates the expression of the FMN riboswitch-containing gene *ribB*^38^. In order to identify other genes whose expression is affected in response to riboflavin, we performed RNAseq in *V. cholerae* N16961 cultures growing in T minimal medium with or without riboflavin. Also, to start elucidating putative differential roles of the riboflavin provision pathways, we included in this analysis the ∆*ribD* and ∆*ribN* derivative strains. The *V. cholerae* ∆*ribD* is a riboflavin auxotroph unable to grow in T media without riboflavin, while the ∆*ribN* does not has an impairment to grow without riboflavin compared to the WT^45^. A general overview of strains, growth conditions and transcriptomics comparisons is presented in Fig. 1. Four transcriptomics comparisons were performed as follows: WT growing without riboflavin versus WT with riboflavin (Comparison **a** in Fig. 1), WT versus ∆*ribD* both with riboflavin (**b**), WT versus ∆*ribN* both with riboflavin (**c**). The genes showing a difference of at least one fold in expression in any of these comparisons were selected and are shown in Table 1. Additionally, a comparison of ∆*ribN* without riboflavin versus ∆*ribN* with riboflavin (**d**) was performed. Genes showing more than one fold change in this comparison and also found in any of the three previous comparisons are indicated in Table 1. In all cases, the genes selected presented a statistically significant change in expression (P < 0.05).

**Table 1:** List of genes whose expression is affected in response to exogenous riboflavin or deletions of *ribD* or *ribN*.

Gene ID	Gene Name	Gene Description	Fold Change (Log2)		
			WT → WT RF+	WT RF + → Δ*ribD* RF+	WT RF + → Δ*ribN* RF+
VC0010		amino acid ABC transporter periplasmic amino acid-binding portion		1.809	
VC0018	*ibpA*	16 kDa heat shock protein A		−1.740	
VC0027		threonine dehydratase		−1.226	−1.191
VC0028		dihydroxy-acid dehydratase			−1.087
VC0030	*ilvM*	acetolactate synthase II small subunit		1.116	
VC0053		hypothetical protein		1.074	
VC0089		cytochrome c551 peroxidase		1.076	
VC0102		hypothetical protein			−1.160
VC0138		hypothetical protein		−1.564	
VC0139		DPS family protein		−1.561	−1.012
VC0143		hypothetical protein	−1.028		
VC0162		ketol-acid reductoisomerase		1.881	1.161
VC0199		hemolysin secretion ATP-binding protein%2 C putative	−1.428		
VC0200	*fhuA*	OMT ferrichrome	−2.453◊		
**VC0201**	*fhuC*	IMT ferrichrome	−1.308◊		
VC0202		iron(III) ABC transporter%2 C periplasmic iron-compound-binding protein	−1.349◊		
VC0211	*pyrE*	orotate phosphoribosyltransferase		1.218	
VC0216		methyl-accepting chemotaxis protein		1.316	
VC0301		hypothetical protein		−1.087	−1.191
**VC0364**	*bfd*	bacterioferritin-associated ferredoxin	−1.459◊		
**VC0365**	*bfr*	bacterioferritin	−1.124		
VC0366	*rpsF*	ribosomal protein S6			1.152
VC0367		primosomal replication protein N			1.136
VC0368	*rpsR*	ribosomal protein S18			1.099
VC0382		hypothetical protein			1.060
VC0383		hypothetical protein			1.198
VC0384		sulfite reductase (NADPH) flavoprotein alpha-component		1.208	
VC0420		conserved hypothetical protein		−1.050	
VC0426		hypothetical protein		−1.708	
VC0430		immunogenic protein		1.017	
VC0438		conserved hypothetical protein			−1.114
VC0488		extracellular solute-binding protein putative		1.067	
VC0491		hypothetical protein		1.026	
VC0492		hypothetical protein		1.273	
VC0503		conserved hypothetical protein			−1.667
VC0515		conserved hypothetical protein		1.158	
VC0546		hypothetical protein			−1.215
VC0548	*csrA*	carbon storage regulator		−1.264	−1.109
VC0549		hypothetical protein		1.054	
VC0550		oxaloacetate decarboxylase alpha subunit		1.010	
VC0589		ABC transporter ATP-binding protein		−1.010	
VC0607		pseudogene		1.102	
**VC0608**	*fbpA*	Iron(III) ABC transporter	−1.439◊		
VC0625		hypothetical protein			−1.140
VC0633	*ompU*	outer membrane protein OmpU		1.362	−1.261
VC0651		conserved hypothetical protein			−1.750
VC0652		protease putative			−2.054
VC0654		conserved hypothetical protein			−1.471
VC0655		acetyltransferase putative			−1.182
VC0706		sigma-54 modulation protein putative			−1.297
VC0707		hypothetical protein		−1.043	
VC0708	*bamD*	conserved hypothetical protein		−1.124	
VC0711	*clpB*	clpB protein		−2.092	−1.022
VC0734		malate synthase A		3.115	
VC0735		hypothetical protein		3.069	
VC0736		isocitrate lyase		1.788	
VC0748		aminotransferase NifS class V		−1.034	
VC0749		NifU-related protein		−1.254	
VC0750	*hesB*	hesB family protein		−1.166	
VC0753		ferredoxin		−1.008	−1.100
VC0754		conserved hypothetical protein			−1.079
VC0765		conserved hypothetical protein			−1.475
**VC0771**	*vibB*	vibriobactin-specific isochorismatase	−1.315		
VC0824	*tpx*	tagD protein		1.636	
VC0855	*dnaK*	dnaK protein		−1.560	
VC0856	*dnaJ*	dnaJ protein		−1.504	
VC0863		conserved hypothetical protein		1.115	
VC0878	*rpmE2*	ribosomal protein L31P family			−1.276
VC0879	*rpmJ*	ribosomal protein L36 putative			−1.121
VC0895		hypothetical protein			−1.190
VC0905	*metQ*	D-methionine transport system substrate-binding protein		1.230	
VC1049	*aphB*	transcriptional regulator LysR family			−1.111
VC1075		conserved hypothetical protein			−1.086
VC1077		hypothetical protein			−1.136
VC1091		oligopeptide ABC transporter periplasmic oligopeptide-binding protein		2.133	
VC1114	*bioC*	biotin synthesis protein BioC			−1.556
VC1115	*bioD*	dethiobiotin synthetase			−1.750
VC1117	*htpX*	heat shock protein HtpX		−1.069	
VC1139		phosphoribosyl-AMP cyclohydrolase/phosphoribosyl-ATP pyrophosphohydrolase		1.072	
VC1147		iron-containing alcohol dehydrogenase		1.203	
VC1157		response regulator		1.183	
VC1169	*trpA*	tryptophan synthase alpha subunit		1.028	
VC1175		hypothetical protein			1.153
VC1206	*gntR*	histidine utilization repressor	1.631◊		
VC1217		conserved hypothetical protein		−1.070	
VC1224		hypothetical protein		−1.101	
VC1226		thiopurine methyltransferase			−1.344
VC1227		hypothetical protein			−1.250
VC1235		sodium/dicarboxylate symporter		1.325	
VC1248		methyl-accepting chemotaxis protein			1.355
**VC1264**	*irpA*	fuction unknown, COG3487	−1.406◊		
**VC1266**		hypothetical periplasmic lipoprotein, like to irpA, COG3488	−1.086		
VC1278		transcriptional regulator MarR family		2.100	
VC1279		transporter BCCT family		4.896	
VC1280		hypothetical protein		1.144	
VC1314		transporter putative		1.487	
VC1315		sensor histidine kinase		1.179	
VC1324		hypothetical protein			1.104
VC1343		peptidase M20A family			−1.335
VC1373		DnaK-related protein		−1.039	
VC1386		chaperone		−1.079	
VC1414	*taq*	thermostable carboxypeptidase 1		1.145	
VC1489		hypothetical protein		−1.609	−1.454
VC1510		hypothetical protein		1.168	1.016
VC1511		formate dehydrogenase cytochrome B556 subunit		1.521	1.102
VC1512		formate dehydrogenase iron-sulfur subunit		1.604	1.100
VC1513		pseudogene		2.147	1.251
VC1514		hypothetical protein		2.306	1.395
VC1515		chaperone formate dehydrogenase-specific putative		2.761	1.920
VC1516		iron-sulfur cluster-binding protein		2.750	2.064
VC1517		hypothetical protein		1.484	1.143
VC1518		hypothetical protein		1.735	1.252
VC1523		conserved hypothetical protein		1.852	1.043
VC1524		ABC transporter permease protein		1.617	
**VC1547**	*exbB*	exbB related linked to tonB2	−1.006		
**VC1548**		hypothetical, linked to tonB2	−1.083		
VC1551		glycerol-3-phosphate ABC transporter permease protein		−1.055	
VC1559		hypothetical protein		−1.371	
VC1560		catalase/peroxidase		−1.450	
VC1563		conserved hypothetical protein			1.068
VC1564		hypothetical protein			1.155
VC1565	*tolC*	outer membrane protein TolC putative			1.202
VC1581	*nuoL*	NADH dehydrogenase putative		2.736	
VC1582		conserved hypothetical protein		1.969	
**VC1688**		hypothetical protein	−1.127		
VC1704	*metE*	5-methyltetrahydropteroyltriglutamate–homocysteine methyltransferase		3.435	
VC1719	*torR*	DNA-binding response regulator TorR			−1.718
VC1731		conserved hypothetical protein			−1.084
VC1808		hypothetical protein		1.396	
VC1823	*fruA*	PTS system fructose-specific IIB component		1.385	
VC1865		hypothetical protein			−1.376
VC1871		conserved hypothetical protein			−1.034
VC1949	*pvcA*	pvcA protein		1.021	
VC1950		biotin sulfoxide reductase			−1.785
VC1951	*yecK*	cytochrome c-type protein YecK			−1.854
VC1956	*mltB*	lytic murein transglycosylase putative		−1.242	
VC1957		conserved hypothetical protein		−1.314	
VC1958		hypothetical protein		−1.144	
VC1962		lipoprotein		−1.070	−1.215
VC1971	*menE*	o-succinylbenzoic acid–CoA ligase		1.181	
VC1972	*menA*	o-succinylbenzoate-CoA synthase			−1.587
**VC1973**	*menB*	naphthoate synthase			−2.445
VC1974	*menH*	conserved hypothetical protein			−2.129
VC2001	*yeaD*	conserved hypothetical protein		1.019	
VC2007		transcriptional regulator ROK family		1.118	
VC2013	*ptsG*	PTS system glucose-specific IIBC component		1.038	
VC2036	*asd*	aspartate-semialdehyde dehydrogenase		1.069	
VC2045	*sodA*	superoxide dismutase Fe		−1.249	−1.328
VC2051	*ccmG*	cytochrome c biogenesis protein			−1.131
VC2052	*ccmF*	cytochrome c-type biogenesis protein CcmF			−1.306
VC2053	*ccmE*	cytochrome c-type biogenesis protein CcmE			−1.828
VC2054	*ccmD*	heme exporter protein D			−1.708
VC2055	*ccmC*	heme exporter protein C			−1.490
**VC2076**	*feoC*	putative ferrous iron transport protein C	−1.421◊		
**VC2077**	*feoB*	ferrous iron transport protein B	−1.489◊		
**VC2078**	*feoA*	ferrous iron transport protein A	−1.172		
VC2149		hypothetical protein			−1.007
VC2174	*ushA*	UDP-sugar hydrolase		1.318	
VC2221		hypothetical protein		1.443	
VC2271	*ribD*	riboflavin-specific deaminase		−1.385	
VC2272	*nrdR*	conserved hypothetical protein		1.858	
VC2323		conserved hypothetical protein		−1.227	
VC2352		NupC family protein		1.381	1.164
VC2357		hypothetical protein		1.362	
VC2361	*grcA*	formate acetyl transferase-related protein	1.163◊		1.092
VC2363	*thrB*	homoserine kinase		1.009	
VC2364	*thrA*	aspartokinase I/homoserine dehydrogenase threonine-sensitive		1.391	
VC2367		hypothetical protein		−1.123	
VC2368	*arcA*	aerobic respiration control protein FexA			−1.409
VC2371		conserved hypothetical protein			−1.303
VC2372		hypothetical protein			−1.395
VC2373	*gltD*	glutamate synthase large subunit		1.126	
VC2417	*recJ*	single-stranded-DNA-specific exonuclease RecJ		−1.098	
VC2418	*dsbC*	thiol:disulfide interchange protein DsbC		−1.200	
VC2419	*xerD*	integrase/recombinase XerD		−1.173	
VC2466	*rseA*	sigma-E factor negative regulatory protein RseA		−1.130	
VC2486		hypothetical protein		−1.035	
VC2490	*leuA*	2-isopropylmalate synthase		1.135	
VC2508	*argF*	ornithine carbamoyltransferase		−1.487	
VC2509		hypothetical protein		−1.032	
VC2510	*pyrB1*	aspartate carbamoyltransferase catalytic subunit		1.319	
VC2511	*pyrB2*	aspartate carbamoyltransferase regulatory subunit		1.394	
VC2524	*ksdC*	conserved hypothetical protein		−1.199	
VC2543		hypothetical protein		1.076	
VC2544	*fbp*	fructose-16-bisphosphatase		1.614	
VC2560	*cysN*	sulfate adenylate transferase subunit 2		1.463	
VC2562	*cpdB*	2′3′-cyclic-nucleotide 2′-phosphodiesterase		1.206	
VC2565	*elaA*	elaA protein			−1.108
VC2568	*fklB*	peptidyl-prolyl cis-trans isomerase FKBP-type		1.042	
VC2637		peroxiredoxin family protein/glutaredoxin		−1.378	
VC2644	*argC*	N-acetyl-gamma-glutamyl-phosphate reductase		−1.289	
VC2645	*argE*	acetylornithine deacetylase		−1.080	
VC2656	*frdA*	fumarate reductase flavoprotein subunit		1.103	
VC2657	*frdB*	fumarate reductase iron-sulfur protein		1.360	
VC2658	*frdC*	fumarate reductase 15 kDa hydrophobic protein		1.708	
VC2659	*frdD*	fumarate reductase 13 kDa hydrophobic protein		1.699	
VC2674	*hslU*	protease HslVU ATPase subunit HslU		−1.330	
VC2675	*hslV*	protease HslVU subunit HslV		−1.258	
VC2689	*pfkA*	6-phosphofructokinase isozyme I			−1.076
VC2699	*dcuA*	C4-dicarboxylate transporter anaerobic		1.040	
VC2706		conserved hypothetical protein		1.577	1.529
VC2720	*nfuA*	conserved hypothetical protein		−1.197	−1.084
VC2738	*pckA*	phosphoenolpyruvate carboxykinase		1.086	
VCA0011	*malT*	malT regulatory protein		1.882	
VCA0013	*malP*	maltodextrin phosphorylase		1.713	
VCA0014	*malQ*	4-alpha-glucanotransferase		1.698	
VCA0015		hypothetical protein		1.630	
VCA0016		14-alpha-glucan branching enzyme		1.642	
VCA0025		transporter NadC family		1.244	
VCA0053	*ppnP*	purine nucleoside phosphorylase			1.062
VCA0087		hypothetical protein			−1.004
VCA0139		hypothetical protein	−1.146		−1.236
VCA0180	*pepT*	peptidase T			−1.364
VCA0205		C4-dicarboxylate transporter anaerobic		1.170	1.136
**VCA0216**		hypothetical membrane, linked to VCA0215 and VCA0217	−1.395		
**VCA0231**	*vctR*	linked to vctA, function unknown	−1.327		
VCA0245	*cmtB*	PTS system IIA component		1.105	
VCA0246	*sgaT*	SgaT protein		1.073	
VCA0268		methyl-accepting chemotaxis protein	−1.056		1.152
VCA0269		decarboxylase group II			1.218
VCA0344		hypothetical protein		1.012	
VCA0511	*nrdG*	anaerobic ribonucleoside-triphosphate reductase		1.175	
VCA0516	*ptsIIB*	PTS system fructose-specific IIBC component		2.838	
VCA0517	*fruK*	1-phosphofructokinase		1.948	−1.919
VCA0518	*ptsIIA*	PTS system fructose-specific IIA/FPR component		1.778	−1.113
VCA0523	*cqsA*	aminotransferase class II		2.585	
VCA0540		formate transporter 1 putative		−2.633	−4.612
VCA0550		hypothetical protein		−1.096	
VCA0551		hypothetical protein		−1.394	
VCA0592	*nudG*	MutT/nudix family protein		1.661	
VCA0621		transcriptional regulator SorC family			−1.283
VCA0628		SecA-related protein		1.536	
VCA0665	*dcuC*	C4-dicarboxylate transporter anaerobic			−1.512
VCA0721		hypothetical protein			−1.014
VCA0752	*trx2*	thioredoxin 2		−1.252	
VCA0773		methyl-accepting chemotaxis protein		1.209	
VCA0784		hypothetical protein			−1.566
VCA0819	*groES*	chaperonin 10 Kd subunit		−1.227	
VCA0820	*groEL*	chaperonin 60 Kd subunit		−1.119	
VCA0821		hypothetical protein		−1.116	
VCA0823	*ectC*	ectoine synthase		1.304	
VCA0824	*ectB*	diaminobutyrate–pyruvate aminotransferase		1.820	
VCA0825	*ectA*	L-24-diaminobutyric acid acetyltransferase		1.691	
VCA0867	*ompW*	outer membrane protein OmpW		1.639	
VCA0897	*devB*	devB protein		−1.127	
VCA0898	*gnd*	6-phosphogluconate dehydrogenase decarboxylating		−1.401	−1.262
**VCA0907**	*hutZ*	heme binding		−1.430	−1.047
**VCA0908**	*hutX*	Unknown, linked to hutZ		−1.626	−1.091
**VCA0909**	*hutW*	unknown, linked to hutZ	−3.049◊		
**VCA0910**	*tonB1*	tonB1 protein	−3.208◊		
**VCA0911**	*exbB1*	TonB system transport protein ExbB1	−3.328◊		
**VCA0912**	*exbD1*	TonB system transport protein ExbD1	−2.996◊	2.023	
**VCA0913**	*hutB1*	hemin ABC transporter%2 C periplasmic hemin-binding protein HutB	−2.383◊		
**VCA0914**	*hutB2*	hemin ABC transporter%2 C permease protein%2 C putative	−1.808◊		
VCA0944	*malF*	maltose ABC transporter permease protein		1.853	
VCA0945	*malE*	maltose ABC transporter periplasmic maltose-binding protein		1.986	
VCA0954	*cheV*	chemotaxis protein CheV putative			−1.029
VCA0965		GGDEF family protein		−1.396	
VCA0966		hypothetical protein		−1.335	
VCA0967		hypothetical protein		−1.507	−1.135
VCA0968		hypothetical protein		−1.527	−1.190
VCA0979		methyl-accepting chemotaxis protein			1.006
VCA0981		hypothetical protein			1.008
VCA0985		oxidoreductase/iron-sulfur cluster-binding protein		−1.381	
VCA0988		methyl-accepting chemotaxis protein	−1.119		
VCA1006		organic hydroperoxide resistance protein putative		−1.130	
VCA1007		hypothetical protein		−1.064	
VCA1009		hypothetical protein			−1.260
VCA1010		conserved hypothetical protein			−3.403
VCA1014		hypothetical protein			1.080
VCA1027	*malM*	maltose operon periplasmic protein putative		1.060	
VCA1028	*lamB*	maltoporin		2.485	
VCA1060	*ribB*	34-dihydroxy-2-butanone 4-phosphate synthase	−3.58	1.476	2.938
VCA1063	*speC*	ornithine decarboxylase inducible			1.067
VCA1064		hypothetical protein			1.366
VCA1069		methyl-accepting chemotaxis protein		1.383	
VCA1099		ABC transporter permease protein		1.081	

The genes with at least one fold change in expression and statistical significance (P < 0.05) are shown. Bold gene IDs indicates genes regulated by iron as reported in ref.^46^ RF, riboflavin. ^◊^Genes with expression affected by riboflavin also in the *∆ribN* strain (comparison **d** in Fig. 1).

**Figure 2:**
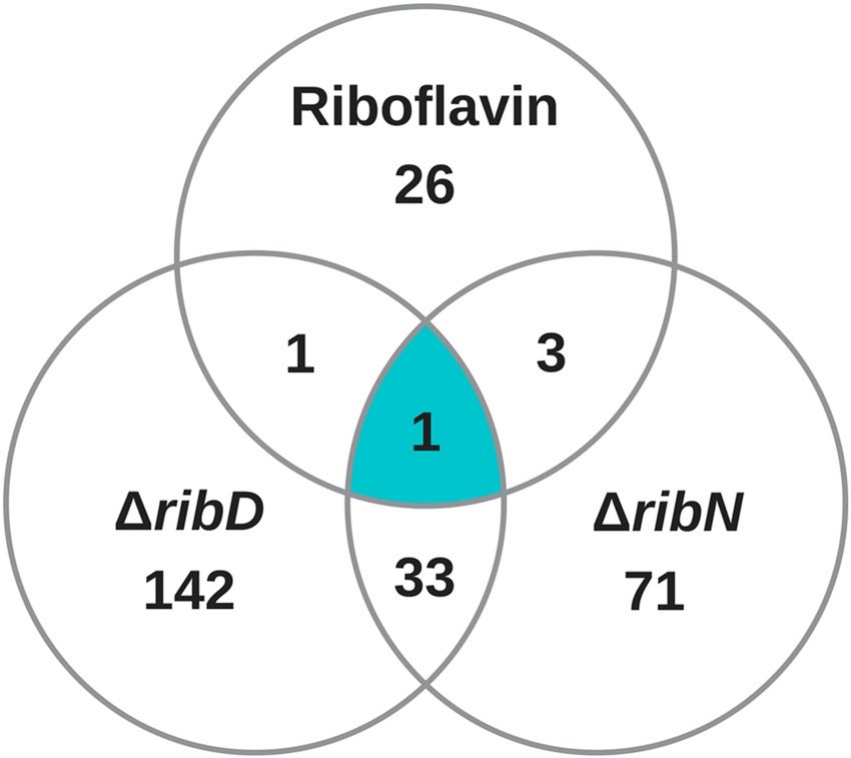
Summary of the results of transcriptomics comparisons. Venn diagram showing the distribution of the genes affected by exogenous riboflavin, the deletion of the riboflavin biosynthetic gene *ribD* and the deletion of the riboflavin transporter gene *ribN*, as determined by transcriptomics.

A total of 277 genes are differentially expressed in response to at least one of the three first conditions compared (Table 1). The results of the indicated comparisons is summarized as a Venn diagram in Fig. 2. 31 regulated genes were differentially expressed in the WT strain in response to extracellular riboflavin (Table 1). 177 genes were significantly affected by the mutation in *ribD*, of which 34 were also affected in the *ribN* mutant. A total of 108 genes were affected by the elimination of *ribN* growing in riboflavin, 74 of which were not affected by the *ribD* elimination. One gene was affected in the three comparisons, which corresponded to the FMN riboswitch-regulated *ribB* (VCA1060). These data are consistent with the notion that although the functions of riboflavin biosynthesis and transport through RibN overlap, there may also exist specific functions for each riboflavin provision pathway.

### The riboflavin regulon of *V. cholerae* includes many iron regulated genes

The first transcriptomic comparison assessed the effect of riboflavin in the WT strain. The gene *ribB* was found at the top of the list of genes regulated by riboflavin, being highly repressed. This pattern is consistent with our previous report, although the degree of repression (roughly 12-fold) was higher than in our earlier determination (2-fold decrease)^38^. A previous RNA microarray study identified 84 genes regulated by iron in *V. cholerae*^46^. Most of the genes identified here as responding to riboflavin, are also members of such iron regulon (21 out of 31). *V. cholerae* possesses several transport systems dedicated to the uptake of various iron forms. These include the genes for synthesis and utilization of the vibriobactin siderophore, the ferrous iron transport system FeoABC, the ferric iron acquisition system FbpABC, the Hut heme transport and the VctPDGC system^47–49^. Likely, these systems are differentially required depending on the iron source available on each stage of the *V. cholerae* life cycle^49^. Genes related to most of these iron acquisition systems, except for the VctPDGC system, were found to be moderately repressed by riboflavin (Table 1), while all of such systems are known to be repressed by iron^46,48^. Other genes belonging to the iron regulon that were also detected responding to extracellular riboflavin included the bacterioferritin operon *bfd-bfr* and a few proteins with unknown function like the encoded by the VC1264, VC1266 and VC0143 open reading frames (ORFs). In addition, *ybtA*, coding for a member of the AraC family of transcriptional regulators involved in regulation of siderophore production in *Yersinia*^50^, was also repressed by riboflavin. Genes identified here which have not previously reported to be regulated by iron include *hutC*, coding for a transcriptional regulator of the histine utilization operon, *glcA*, coding for the autonomous glycyl radical cofactor protein and two methyl-accepting chemotaxis protein genes. While most of the genes identified in this comparison were repressed by riboflavin, *hutC* and *glcA* were activated. In our previous study, contrary to its effect on the WT strain, riboflavin induced the expression of *ribB* in a *∆ribN* strain^45^. This suggested that riboflavin may induce changes in transcription in a manner independent of its internalization through RibN. For this reason, in order to globally identify effects of riboflavin independent of its uptake through RibN, we included a comparison of the transcriptomes in response to riboflavin in the ∆*ribN* strain. This analysis revealed that 16 of the genes affected by riboflavin in the WT are also affected in this strain (indicated by asterisks in Table 1 and full list in Table S1). This suggests that at least for these cases, the regulatory effect of riboflavin is independent of its internalization through RibN. In order to identify general functional relationships among the genes responsive to riboflavin, we performed analysis of enrichment of Gene Ontology (GO) terms of biological processes associated to this set. Such analysis seek to identify functional terms, as defined by the PANTHER classification system, overrepresented in a given group of genes^51,52^. Three GO biological processes were found statistically overrepresented (P < 0.05). These corresponded to *iron ion transmembrane transport*, *cellular responses to iron ion* and *iron ion homeostasis*.

**Figure 3:**
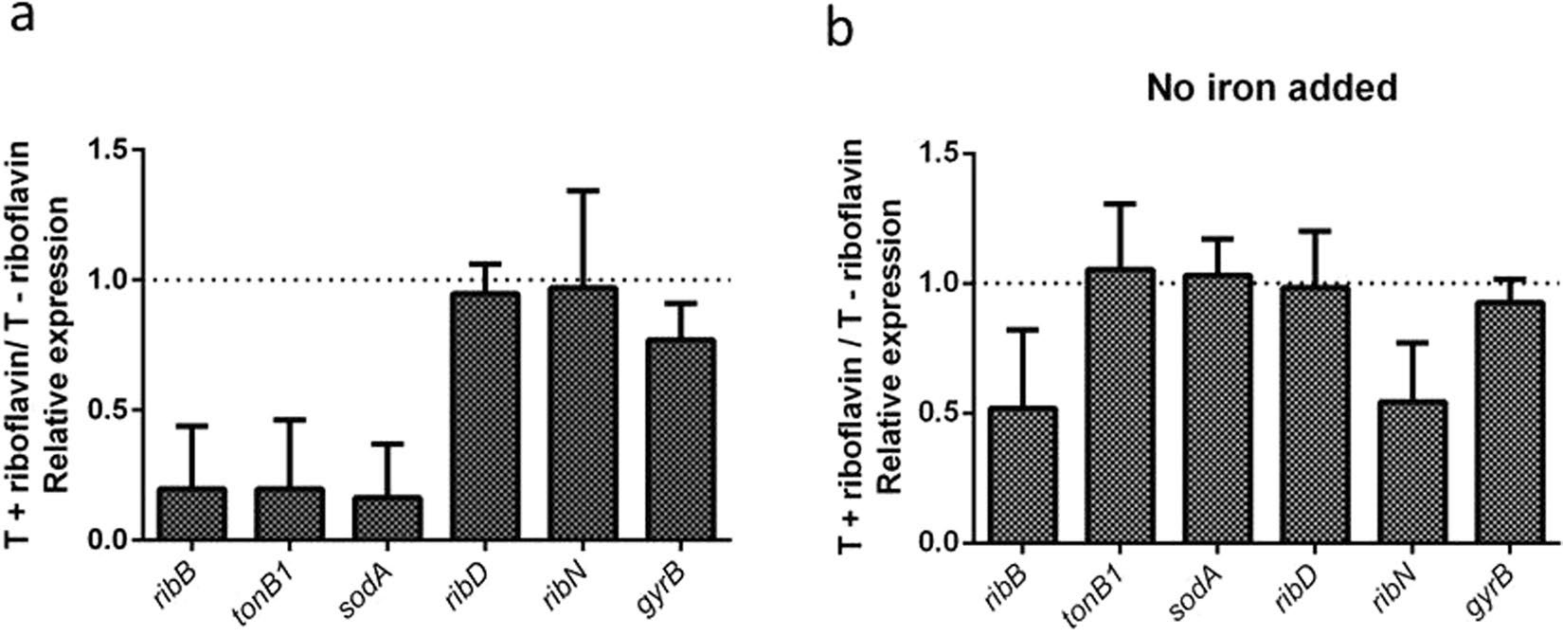
Effect of riboflavin on the expression of genes under different iron conditions. Relative expression of the indicated genes with and without riboflavin in T media (**a**) or T without added iron (**b**), as determined by RT-PCR. WT *V. cholerae* was grown until medium exponential phase at 37 °C, RNA extracted and RT-PCR assayed as described in Materials and Methods. Results shown are the average and standard deviation of three independent experiments.

To validate the transcriptomic comparison, we determined the relative expression of *ribB* and *tonB1* in T medium and T plus riboflavin by RT-PCR. The *tonB1* gene encodes a component of one of the two TonB-ExbB-ExbD complexes that harness membrane proton motive force for its heterologous use in various iron transport systems in *V. cholerae*^47^. Thus, it seems to be an adequate gene to monitor the expression of iron acquisition systems. The expression of *ribB* and *tonB1* was reduced 4-fold in response to added riboflavin (Fig. 3a). This is in agreement with the transcriptomics results although a higher effect of riboflavin was detected by RT-PCR. To assess if there may be additional genes known to be regulated by iron that are also regulated by riboflavin but missed in our transcriptomics analysis, we determined the expression of *sodA*. This gene is known to be repressed by iron^46^. Notably, the expression of *sodA* was reduced 4.13-fold by riboflavin. As controls, we determined the expression of the riboflavin biosynthetic gene *ribD* and of the *ribN* gene. We have previously demonstrated that the expression of these genes is not affected by riboflavin in standard T media^38^ and their expression did not change in response to riboflavin in our transcriptomics results. Accordingly, the expression of these two genes was not affected by riboflavin as determined by RT-PCR. One additional control was used, *gyrB*, which was not affected by the presence of exogenous riboflavin according to transcriptomics. The RNA of this gene was only slighty reduced by riboflavin (0.29-fold) as determined by RT-PCR.

### Riboflavin and iron reciprocally regulate their provision genes

**Figure 4:**
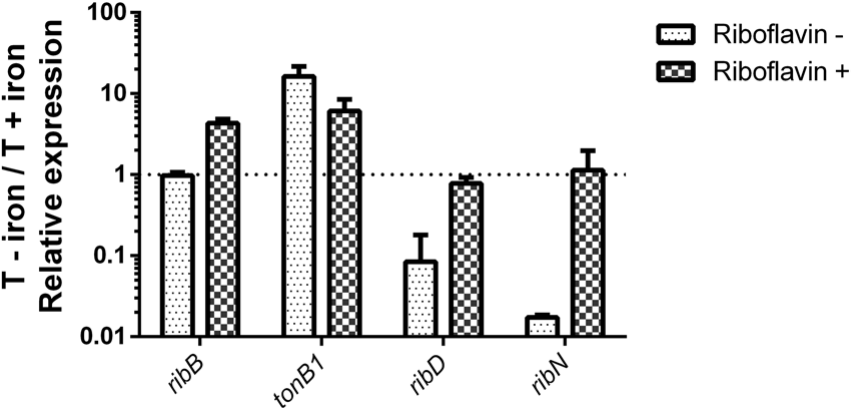
Effect of iron in the expression of genes under different riboflavin conditions. The relative expression of the genes in T without iron versus complete T, with and without riboflavin as indicated. Growth conditions were similar as those described in Fig. 3. Results shown are the average and standard deviation of three independent experiments.

Thus far, results indicate that riboflavin regulates many genes that are also regulated by iron. The experiments were performed in standard T media. The recipe for this medium includes 20 µM FeCl and may be considered an iron-replete condition when compared to minimal media without added iron^46,53^. It is reported that in such conditions, the iron aquisiton systems of *V. cholerae* are mainly repressed^47^. Thus, in the case of iron uptake genes, riboflavin seems to enhance the repression produced in high iron conditions. Along these lines, we aimed to determine the effect of riboflavin on the expression of iron regulated genes under iron-restrictive conditions. These conditions are known to induce the expression of iron uptake systems. For this, we grew *V. cholerae* in T media without any added iron and determined the effect of riboflavin by RT-PCR. Notably, the expression of *tonB1* and *sodA*, as well as that of the controls *ribD* and *gyrB*, remained around the same with or without riboflavin in such iron-restrictive conditions (Fig. 3b). This may indicate that the negative modulatory effect of riboflavin is surpassed under the highly inducing conditions triggered by iron restriction. Strikingly, the expression of *ribN* was diminished by half by riboflavin in this condition, in contrast with the results obtained in iron repleted media, were riboflavin has no effect. This suggests that riboflavin modulates the expression of *ribN* but only during iron restriction. To corroborate that *tonB1* is being induced in response to iron restriction in our experiments, we compared the expression of this gene when growing without iron versus standard T media. We assessed this in media with and without riboflavin. Irrespective of the presence of riboflavin, the expression of *tonB1* is highly increased (more than 10-fold) in low iron media, although the induction effect was higher without riboflavin (Fig. 4). Remarkably, although iron has no effect in the expression of *ribD* when riboflavin is present, in riboflavin-free media this gene was highly repressed in iron-restrictive conditions. The same occurred for the *ribN* gene. Nonetheless, a different effect applied for the *ribB* gene. In the absence of riboflavin, iron had no effect, while iron restriction increased the expression of this gene 3-fold in the presence of exogenous riboflavin. Collectively, results indicate that riboflavin and iron interplay affects the expression of iron and riboflavin provision genes in a gene-specific manner.

### Genes affected both in the *ribD* and *ribN* mutants

We have recently hypothesized that riboflavin transport, instead of merely replacing for the RBP, may afford riboflavin for specific physiological functions^23^. The results of the transcriptomics comparisons performed here show that 34 of the genes whose expression is affected by the elimination of riboflavin biosynthesis are also affected by the elimination of the RibN importer (Table S2). This clearly suggests that functional overlap between riboflavin biosynthesis and internalization occurs. Five of these genes belong to the iron regulon. These are the VC1515, VCA1516, VC1514, VCA0908 and VCA0907 ORFs. VC1514 encodes a protein of unknown function putatively encoded in the same operon as VC1515 and VC1516. The latter genes code for a putative chaperone of a formate dehydrogenase and an iron-sulfur cluster binding protein, respectively. Thus, this system seems to be involved in redox reactions although its exact function is unknown. The transcription of these genes is increased in both *ribD* and *ribN* mutants. VCA908 and VCA907 are encoded within a putative operon that codes for proteins involved in heme utilization. These ORFs were found to be downregulated in response to *ribD* and *ribN* eliminations. The set of genes regulated in the two conditions also included *ribB*, which was upregulated as a result of both mutations. As this gene conserves an FMN riboswitch that represses expression in response to FMN, its induction probably reflects a reduction in intracellular riboflavin levels produced in both mutants. Genes encoding proteins involved in amino acids metabolism, such as VC0162 coding for a ketol-acid reductoisomerase and VC0027, coding for threonine dehydratase, as well as enzymes involved in redox metabolism such as VC2045, coding for a superoxide dismutase and VC0753, encoding a ferredoxin, were also included in this subgroup. An analysis of enrichment of GO terms of biological processes associated to this set rendered no significant overrepresentation.

Most of the genes within this group followed the same pattern of regulation irrespective of whether the elimination was on *ribD* or *ribN*. However, three genes were differentially affected by the mutations. VCA0517 and VCA0518, encoded in an operon of a phosphotransferase system for fructose transport, were upregulated roughly 3.5 times in the ∆*ribD* strain but downregulated 3.7 and 2.1 times respectively in the ∆*ribN* strain. Likewise, the gene for OmpU, one of the major porins in this species, was upregulated 2.5 times in the ∆*ribD* strain but downregulated 2.3 times in the ∆*ribN* strain. These represent an intriguing group, as the transcription of these genes seems to be reciprocally regulated by riboflavin biosynthesis and riboflavin uptake through RibN.

### Genes specifically affected in response to *ribD* elimination

The transcription of 139 genes was significantly affected by the elimination of *ribD* but not by the elimination of *ribN*. This comprised the largest set of genes defined by any of our comparisons (Table S3). The VC1279 ORF, encoding a putative member of the Betaine/Carnitine/Choline Transporters (BCCT) family, was the highest regulated gene, being induced 29.6 times in response to *ribD* elimination. Also atop of the list were the genes VC1704, encoding a 5-methyltetrahydropteroyltriglutamate-homocysteine methyltransferase required for cystein and methionin biosynthesis and VC0734, coding for a malate synthase. The list included some iron regulated genes, such as *exbD1*, related to the TonB1 system and many genes related to the PTS system for fructose and glucose uptake. In the GO terms enrichment analysis for this subset two terms were overrepresented: *protein folding* and *oxidation-reductions process*.

### Genes specifically affected in response to *ribN* mutation

We identified genes whose expression changed in the *ribN* mutant but not in the *ribD* mutant. 73 genes corresponded to this pattern of regulation (Table S4). In this list two genes involved in heme export, VC2054 and VC2055 were downregulated, which could also be related to the riboflavin-iron metabolic interplay highlighted across the transcriptomics results. Notably, many ribosome assembly genes also appeared in this set of genes. Among the most regulated genes are *menB* and *menH*, both involved in menaquinone (vitamin K) biosynthesis, *bioD*, required for biotin biosynthesis, VC1950, which encodes a biotin sulfoxide reductase that allows biotin salvatage and *yecK* and *ccmE*, two genes involved in cytochrome c-type biogenesis. The list included other genes also involved in biotin biosynthesis and cytochrome c-type biogenesis such as *bioC* and *ccmF*, respectively. All of these proteins were downregulated in the *∆ribN* strain. Thus it seems that the lack of transport of riboflavin through RibN downregulates menaquinone, biotin and cytochrome c biosynthesis. Accordingly, the GO term *cytochrome complex assembly* was significantly enriched in this subset of genes. Menaquinone and cytochromes participate in electron transfer chains. Notably, the ArcA response regulator that control aerobic respiration was also downregulated. Thus, these results suggest that internalized riboflavin may be involved in respiratory chain processes in *V. cholerae*.

## Discussion

This study assessed the effect of riboflavin on gene expression in *V. cholerae*. Many of the genes affected by riboflavin are known to be regulated by the iron levels in the media. The determination of the expression of genes by RT-PCR added *sodA* to the list of genes downregulated by riboflavin. Thus, our transcriptomics analysis may be underestimating the number of genes regulated by riboflavin and the overlapping of iron and riboflavin regulons could be more extensive. Genes belonging to five out of six iron acquisition systems present in this species were negatively modulated by the presence of riboflavin in T media. These systems are known to be repressed in iron-rich environments and induced under iron deprivement. When assessed its effect in low iron, riboflavin no longer repressed iron regulated genes. Thus, riboflavin seems to accent a high iron condition in the expression of iron uptake systems and possibly other iron regulated genes, while having no repressive effect during iron starvation. Contrarywise, the riboflavin transport gene *ribN*, which is expressed independently of riboflavin in T media with iron, was negatively modulated by exogenous riboflavin during iron starvation. Reciprocally, iron repressed the expression of *ribD* and *ribN* but only in the absence of exogenous riboflavin, while inducing the expression of *ribB* in the presence of riboflavin. These three genes are encoded in separated transcriptional elements. Noteworthy, *ribB* is the only one conserving a FMN riboswitch^38^, likely rendering the expression of this gene coupled to the levels of intracellular riboflavin. This may be responsible for its differential regulation. The increase in expression of *ribB* in low iron may reflex a decrease in intracellular riboflavin levels. This may seem paradoxical given that this effect only occurs in the presence of extracellular riboflavin. However, we have previously observed an increase in *ribB* transcription in the presence of riboflavin in a *ribN* mutant^38^, and such result replicated in this transcriptomics analysis. This suggested that the presence of extracellular riboflavin increases intracellular riboflavin requirements. Thus, this increase may be exacerbated in low iron conditions, which may explain this result. In general, the expression of iron and riboflavin provision genes was found to be modulated by the presence of both iron and riboflavin in the media in a coordinated fashion. At least in the case of riboflavin provision genes, this regulation is gene-specific. Altogether, this may reflex a paramount regulatory crosstalk between the two most important redox cofactors in nature. The iron-riboflavin interregulatory effect may be common also in other bacteria. RBP genes have been found upregulated under iron-deficiency conditions by high throughput approaches in different bacteria such as *Caulobacter crescentus*^54^, *Methylocystis*^55^ and *Clostridium acetobutylicum*^56^. The physiological relevance of this is unknown. One probable explanation is that in these species the lack of iron could be compensated by enhancing the biosynthesis of riboflavin, another important redox cofactor. Indeed, flavodoxins seem to substitute for ferredoxins in electron transfer reactions under iron starving conditions in different organisms across kingdoms^57–60^. Nonetheless in *V. vulnificus*, a bacterial species philogenetically related to *V. cholerae*, the RBP genes are downregulated under iron restriction^61^, which is a similar effect to what we observed in this study for *ribD* and *ribN*. Our work provided evidence of the reciprocal phenomenon for the first time, in which the availability of riboflavin alters iron metabolism in bacteria. Altogether, the overlay between riboflavin and iron regulons suggests the existence of a network interconnecting riboflavin and iron homeostasis and probably a common regulatory mechanism. This seems an important feature that grants further study.

The way riboflavin biosynthesis and uptake correlate to fulfill the flavin needs in riboflavin opportunistic species is still unclear. Nonetheless, some studies shed light into the role of riboflavin transporters in bacterial physiology. The RibM riboflavin importer is able to provide flavins to a RBP-deficient mutant of *Corynebacterium glutamicum* when growing with extracellular riboflavin, albeit the levels of the intracellular riboflavin pools are lower than those in the WT strain^62^. In *Staphylococcus aureus*, the Energy coupling factor (ECF)-RibU riboflavin uptake system is able to fully substitute for the RBP during *in vitro* growth with riboflavin traces and also during mouse infection^63^. Overexpression of RibU, the substrate-binding component of this system, helps overcome heat stress in *Lactococcus lactis*^64,65^. The RfuABCD riboflavin uptake system in *Borrelia burgdorferi* is required to set an efficient oxidative stress response and for colonization in the murine model^66^. In the case of RibN, it is required for full colonization of pea plant roots at early stages by the riboflavin prototroph *Rhizobium leguminosarum*^40^. In *V. cholerae*, riboflavin biosynthesis is sufficient to grow in river water but RibN provides a competitive advantage^45^. Here, transcriptome comparisons suggest that riboflavin biosynthesis and uptake have common and specific effects in gene transcription, which may be related to functions performed by these two riboflavin provision pathways. Remarkably, GO functional terms were distinctively defined in the subsets affected by each deletion. While *protein folding* and *oxidation-reductions process* were enriched in the genes specifically affected by the lack of riboflavin biosynthesis, *cytochrome complex assembly* was enriched in the set of genes pointedly affected by the *ribN* mutation. Other genes involved in electron transport chain were also affected in the ∆*ribN* specific set. Hence, this study may serve as a start point to characterize cellular functions requiring exogenous riboflavin in this species. Notably, the number of genes affected by the elimination of riboflavin biosynthesis was significantly higher than those affected by the presence of external riboflavin or abrogation of RibN. This may pose that biosynthesized riboflavin is engaged in more physiological functions than exogenous riboflavin. The fact that extracellular riboflavin downregulates the monicistronically encoded *ribB* but does not affects the expression of the main RBP operon on which other *ribB* homolog may be encoded also supports this view^38^. This effect could allow to retain the capacity to fully biosynthesize riboflavin in the presence of exogenous riboflavin. Importantly, the elimination of RibN does not necessarily abolish riboflavin uptake, as the presence of additional riboflavin transport systems has not been experimentally determined in this strain. This could be accomplished by the determination of the levels of riboflavin needed to support growth in a double ∆*ribD*/∆*ribN* strain. However, our attempts to obtain such strain have failed even in the presence of high riboflavin concentrations in the media. Nonetheless, the increase in expression of *ribB* induced by exogenous riboflavin in the *ribN* mutant may suggest that riboflavin is not entering the cell by a different transporter.

Collectively, this study comprises an integral analysis of the response induced by availability of riboflavin in *V. cholerae* on what constitutes, to the best of our knowledge, the first approach to a riboflavin regulon in bacteria.

## Electronic supplementary material

Supplementary Dataset 1
