# Supplementary material for: Transcriptomics reveals a cross-modulatory effect between riboflavin and iron and outlines responses to riboflavin biosynthesis and uptake in Vibrio cholerae
Source: Sci Rep. 2018 Feb 16;8:3149. doi: 10.1038/s41598-018-21302-3 (PMC5816637; doi:10.1038/s41598-018-21302-3)
Supplement: Supplementary file 1 — Supplementary Dataset 1 [file 41598_2018_21302_MOESM1_ESM.docx]

**Transcriptomics reveals a cross-modulatory effect between riboflavin and iron and outlines responses to riboflavin biosynthesis and uptake in *Vibrio cholerae***

Ignacio Sepúlveda-Cisternas^1,2*^, Luis Lozano Aguirre^3*^, Andrés Fuentes Flores^1^, Ignacio Vásquez Solis de Ovando^1^ and Víctor Antonio García-Angulo^1&^.

^1^Programa de Microbiología y Micología, Instituto de Ciencias Biomédicas, Universidad de Chile. Santiago, Chile. ^2^Escuela de Biotecnología, Universidad Mayor, Campus Huechuraba, Santiago, Chile Programa de Microbiología y Micología, Instituto de Ciencias Biomédicas, Universidad de Chile. Santiago, Chile. ^3^Centro de Ciencias Genómicas, Universidad Nacional Autónoma de México, campus Chamilpa. Cuernavaca, Morelos, México.

**Supplementary Dataset 1. Genes differentially expressed in the transcriptomics comparisons perfomed**

| **Table S1. .** **Genes affected in response to riboflavin in the *V. cholerae* Δ*ribN* strain** | | | |
| --- | --- | --- | --- |
| **Gene**  **ID** | **Gene**  **Name** | **Gene Description** | **Fold Change (Log 2)** |
| VC0023 |  | NADH dehydrogenase subunit II-related protein | -1.3261 |
| VC0076 | *uspA* | universal stress protein A | -1.2487 |
| VC0438 |  | hypothetical protein | 1.2018 |
| VC0546 |  | hypothetical protein | 1.1804 |
| VC0547 |  | aspartate kinase | 1.0692 |
| VC0651 |  | conserved hypothetical protein | 1.5848 |
| VC0652 |  | putative protease | 1.5600 |
| VC0654 |  | conserved hypothetical protein | 1.5898 |
| VC0655 |  | putative acetyltransferase | 1.3571 |
| VC0734 |  | malate synthase A | 1.3203 |
| VC0735 |  | hypothetical protein | 1.4954 |
| VC0765 |  | conserved hypothetical protein | 1.3070 |
| VC0957 |  | conserved hypothetical protein | -1.1698 |
| VC0972 |  | porin putative | -1.1816 |
| VC1049 | *aphB* | transcriptional regulator LysR family | 1.1369 |
| VC1114 | *bioC* | biotin synthesis protein BioC | 1.2135 |
| VC1115 | *bioD* | dethiobiotin synthetase | 1.3539 |
| VC1153 |  | conserved hypothetical protein | -1.0682 |
| VC1171 | *trpC/trpF* | indole-3-glycerol phosphate synthase/phosphoribosylanthranilate isomerase | -1.2365 |
| VC1172 | *trpD* | anthranilate phosphoribosyltransferase | -1.1432 |
| VC1173 | *trpG* | anthranilate synthase component II | -1.3511 |
| VC1174 | *trpE* | anthranilate synthase component I | -1.4563 |
| VC1175 |  | hypothetical protein | -1.4785 |
| VC1267 |  | hypothetical protein | 1.0282 |
| VC1300 | *sdaA* | L-serine dehydratase 1 | 1.4586 |
| VC1315 |  | sensor histidine kinase | 1.2023 |
| VC1343 |  | peptidase M20A family | 1.4401 |
| VC1472 |  | hypothetical protein | 1.0177 |
| VC1513 |  | pseudogene | 1.0331 |
| VC1514 |  | hypothetical protein | 1.2846 |
| VC1515 |  | chaperone formate dehydrogenase-specific putative | 1.1674 |
| VC1581 | *nuoL* | NADH dehydrogenase putative | 1.5181 |
| VC1582 |  | conserved hypothetical protein | 1.0191 |
| VC1687 | *ppaC* | conserved hypothetical protein | 1.5409 |
| VC1719 | *torR* | DNA-binding response regulator TorR | 1.0190 |
| VC1728 |  | hypothetical protein | -1.2972 |
| VC1731 |  | conserved hypothetical protein | 1.3391 |
| VC1865 |  | hypothetical protein | 1.3197 |
| VC1871 |  | conserved hypothetical protein | 1.3270 |
| VC1950 |  | biotin sulfoxide reductase | 1.6799 |
| VC1951 | *yecK* | cytochrome c-type protein YecK | 1.6634 |
| VC1971 | *menE* | o-succinylbenzoic acid--CoA ligase | 1.7310 |
| VC1972 | *menA* | o-succinylbenzoate-CoA synthase | 2.2701 |
| VC1973 | *menB* | naphthoate synthase | 2.0346 |
| VC1974 | *menH* | conserved hypothetical protein | 1.8368 |
| VC2049 | *ccmI* | cytochrome C biogenesis protein CcmI | 1.3470 |
| VC2051 | *ccmG* | cytochrome c biogenesis protein CcmG | 1.5320 |
| VC2052 | *ccmF* | cytochrome c-type biogenesis protein CcmF | 1.1189 |
| VC2053 | *ccmE* | cytochrome c-type biogenesis protein CcmE | 1.1518 |
| VC2054 | *ccmD* | heme exporter protein D | 1.1500 |
| VC2070 |  | phosphohistidine phosphatase | -1.1096 |
| VC2145 | *tyrA* | tyrA protein | 1.2820 |
| VC2221 |  | hypothetical protein | 1.0931 |
| VC2371 |  | conserved hypothetical protein | 1.1424 |
| VC2372 |  | hypothetical protein | 1.2648 |
| VC2484 | *fadD* | long-chain-fatty-acid--CoA ligase putative | 1.0236 |
| VC2656 | *frdA* | fumarate reductase flavoprotein subunit | 1.3697 |
| VC2657 | *frdB* | fumarate reductase iron-sulfur protein | 1.3532 |
| VC2658 | *frdC* | fumarate reductase 15 kDa hydrophobic protein | 1.2179 |
| VC2659 | *frdD* | fumarate reductase 13 kDa hydrophobic protein | 1.1226 |
| VC2689 | *pfkA* | 6-phosphofructokinase isozyme I | 1.0133 |
| VC2702 |  | transcriptional regulator LuxR family | -1.0350 |
| VCA0011 | *malT* | malT regulatory protein | 1.2371 |
| VCA0053 | *ppnP* | purine nucleoside phosphorylase | -1.0768 |
| VCA0180 | *pepT* | peptidase T | 1.7218 |
| VCA0205 |  | C4-dicarboxylate transporter anaerobic | -1.4540 |
| VCA0507 | *orfA* | transposase OrfAB subunit A | -1.0799 |
| VCA0536 |  | conserved hypothetical protein | 1.0666 |
| VCA0540 |  | formate transporter 1 putative | 3.2628 |
| VCA0592 | *nudG* | MutT/nudix family protein | 1.2488 |
| VCA0621 |  | transcriptional regulator SorC family | 1.1076 |
| VCA0665 | *dcuC* | C4-dicarboxylate transporter anaerobic | 1.9853 |
| VCA0743 |  | conserved hypothetical protein | -1.1061 |
| VCA0784 |  | hypothetical protein | 1.3193 |
| VCA0798 |  | CbbY family protein | -1.4371 |
| VCA1098 |  | ABC transporter periplasmic substrate-binding protein | 1.1860 |

| **Table S2. Genes affected by the elimination of both *ribD* and *ribN.*** | | | | |
| --- | --- | --- | --- | --- |
|  | | | **Fold Change (Log2)** | |
| **Gene**  **ID** | **Gene**  **Name** | **Gene Description** | **WT RF+ →Δ*ribD* RF+** | **WT RF+ →Δ*ribN* RF+** |
| VC0027 |  | threonine dehydratase | -1.2260 | -1.1912 |
| VC0139 |  | DPS family protein | -1.5605 | -1.0124 |
| VC0162 |  | ketol-acid reductoisomerase | 1.8808 | 1.1612 |
| VC0301 |  | hypothetical protein | -1.0873 | -1.1905 |
| VC0548 |  | carbon storage regulator | -1.2637 | -1.1092 |
| VC0633 | *ompU* | outer membrane protein OmpU | 1.3623 | -1.2612 |
| VC0711 | *clpB* | clpB protein | -2.0921 | -1.0218 |
| VC0753 |  | ferredoxin | -1.0081 | -1.1002 |
| VC1489 |  | hypothetical protein | -1.6092 | -1.4536 |
| VC1510 |  | hypothetical protein | 1.1677 | 1.0161 |
| VC1511 |  | formate dehydrogenase cytochrome B556 subunit | 1.5213 | 1.1017 |
| VC1512 |  | formate dehydrogenase iron-sulfur subunit | 1.6040 | 1.1005 |
| VC1513 |  | *pseudogene* | 2.1471 | 1.2513 |
| VC1514 |  | *hypothetical protein* | 2.3063 | 1.3946 |
| VC1515 |  | chaperone formate dehydrogenase-specific putative | 2.7610 | 1.9203 |
| VC1516 |  | iron-sulfur cluster-binding protein | 2.7500 | 2.0642 |
| VC1517 |  | hypothetical protein | 1.4837 | 1.1427 |
| VC1518 |  | hypothetical protein | 1.7348 | 1.2525 |
| VC1523 |  | conserved hypothetical protein | 1.8516 | 1.0431 |
| VC1962 |  | lipoprotein | -1.0696 | -1.2151 |
| VC2045 | *sodA* | superoxide dismutase Fe | -1.2490 | -1.3277 |
| VC2352 |  | NupC family protein | 1.3807 | 1.1643 |
| VC2706 |  | conserved hypothetical protein | 1.5765 | 1.5285 |
| VC2720 | *nfuA* | conserved hypothetical protein | -1.1968 | -1.0842 |
| VCA0205 |  | C4-dicarboxylate transporter anaerobic | 1.1701 | 1.1357 |
| VCA0517 | *fruK* | 1-phosphofructokinase | 1.9480 | -1.9189 |
| VCA0518 | *ptsIIA* | PTS system fructose-specific IIA/FPR component | 1.7782 | -1.1128 |
| VCA0540 |  | formate transporter 1 putative | -2.6333 | -4.6117 |
| VCA0898 | *gnd* | 6-phosphogluconate dehydrogenase decarboxylating | -1.4014 | -1.2624 |
| VCA0907 | *hutZ* | heme binding | -1.4301 | -1.0475 |
| VCA0908 | *hutX* | Unknown, linked to hutZ | -1.6259 | -1.0911 |
| VCA0967 |  | hypothetical protein | -1.5068 | -1.1353 |
| VCA0968 |  | hypothetical protein | -1.5273 | -1.1900 |
| VCA1060 | *ribB* | 34-dihydroxy-2-butanone 4-phosphate synthase | 1.4759 | 2.9377 |

| **Table S3. Genes specifically affected by the *ribD* deletion.** | | | |
| --- | --- | --- | --- |
| **Gene**  **ID** | **Gene**  **Name** | **Gene Description** | **Fold Change (Log2)** |
| VC1279 | *betP* | transporter BCCT family | 4.8959 |
| VC0010 |  | amino acid ABC transporter periplasmic amino acid-binding portion | 1.8087 |
| VC0018 | *ibpA* | 16 kDa heat shock protein A | -1.7401 |
| VC0030 | *ilvM* | acetolactate synthase II small subunit | 1.1163 |
| VC0053 |  | hypothetical protein | 1.0743 |
| VC0089 | *c551* | cytochrome c551 peroxidase | 1.0762 |
| VC0138 |  | hypothetical protein | -1.5639 |
| VC0211 | *pyrE* | orotate phosphoribosyltransferase | 1.2176 |
| VC0216 |  | methyl-accepting chemotaxis protein | 1.3159 |
| VC0384 | *cysJ* | sulfite reductase (NADPH) flavoprotein alpha-component | 1.2081 |
| VC0420 |  | conserved hypothetical protein | -1.0495 |
| VC0426 |  | hypothetical protein | -1.7080 |
| VC0430 |  | immunogenic protein | 1.0174 |
| VC0488 |  | extracellular solute-binding protein putative | 1.0674 |
| VC0491 |  | hypothetical protein | 1.0263 |
| VC0492 |  | hypothetical protein | 1.2730 |
| VC0515 |  | conserved hypothetical protein | 1.1581 |
| VC0549 |  | hypothetical protein | 1.0539 |
| VC0550 |  | oxaloacetate decarboxylase alpha subunit | 1.0102 |
| VC0589 |  | ABC transporter ATP-binding protein | -1.0103 |
| VC0607 |  | pseudogene | 1.1024 |
| VC0707 |  | hypothetical protein | -1.0426 |
| VC0708 | *bamD* | conserved hypothetical protein | -1.1244 |
| VC0734 |  | malate synthase A | 3.1150 |
| VC0735 |  | hypothetical protein | 3.0693 |
| VC0736 |  | isocitrate lyase | 1.7878 |
| VC0748 |  | aminotransferase NifS class V | -1.0339 |
| VC0749 |  | NifU-related protein | -1.2545 |
| VC0750 | *hesB* | hesB family protein | -1.1665 |
| VC0824 | *tpx* | tagD protein | 1.6365 |
| VC0855 | *dnaK* | dnaK protein | -1.5602 |
| VC0856 | *dnaJ* | dnaJ protein | -1.5043 |
| VC0863 |  | conserved hypothetical protein | 1.1149 |
| VC0905 | *metQ* | D-methionine transport system substrate-binding protein | 1.2297 |
| VC1091 |  | oligopeptide ABC transporter periplasmic oligopeptide-binding protein | 2.1325 |
| VC1117 | *htpX* | heat shock protein HtpX | -1.0693 |
| VC1139 |  | phosphoribosyl-AMP cyclohydrolase/phosphoribosyl-ATP pyrophosphohydrolase | 1.0718 |
| VC1147 |  | iron-containing alcohol dehydrogenase | 1.2034 |
| VC1157 |  | response regulator | 1.1825 |
| VC1169 | *trpA* | tryptophan synthase alpha subunit | 1.0276 |
| VC1217 |  | conserved hypothetical protein | -1.0699 |
| VC1224 |  | hypothetical protein | -1.1008 |
| VC1235 |  | sodium/dicarboxylate symporter | 1.3248 |
| VC1278 |  | transcriptional regulator MarR family | 2.1002 |
| VC1280 |  | hypothetical protein | 1.1436 |
| VC1314 |  | transporter putative | 1.4872 |
| VC1315 |  | sensor histidine kinase | 1.1792 |
| VC1373 |  | DnaK-related protein | -1.0394 |
| VC1386 |  | chaperone | -1.0794 |
| VC1414 | *taq* | thermostable carboxypeptidase 1 | 1.1451 |
| VC1524 |  | ABC transporter permease protein | 1.6169 |
| VC1551 |  | glycerol-3-phosphate ABC transporter permease protein | -1.0547 |
| VC1559 |  | hypothetical protein | -1.3707 |
| VC1560 |  | catalase/peroxidase | -1.4500 |
| VC1581 | *nuoL* | NADH dehydrogenase putative | 2.7358 |
| VC1582 |  | conserved hypothetical protein | 1.9690 |
| VC1704 | *metE* | 5-methyltetrahydropteroyltriglutamate--homocysteine methyltransferase | 3.4349 |
| VC1808 |  | hypothetical protein | 1.3958 |
| VC1823 | *fruA* | PTS system fructose-specific IIB component | 1.3845 |
| VC1949 | *pvcA* | pvcA protein | 1.0210 |
| VC1956 | *mltB* | lytic murein transglycosylase putative | -1.2419 |
| VC1957 |  | conserved hypothetical protein | -1.3138 |
| VC1958 |  | hypothetical protein | -1.1436 |
| VC1971 | *menE* | o-succinylbenzoic acid--CoA ligase | 1.1809 |
| VC2001 | *yeaD* | conserved hypothetical protein | 1.0190 |
| VC2007 |  | transcriptional regulator ROK family | 1.1178 |
| VC2013 | *ptsG* | PTS system glucose-specific IIBC component | 1.0378 |
| VC2036 | *asd* | aspartate-semialdehyde dehydrogenase | 1.0686 |
| VC2174 | *ushA* | UDP-sugar hydrolase | 1.3182 |
| VC2221 |  | hypothetical protein | 1.4430 |
| VC2271 | *ribD* | riboflavin-specific deaminase | -1.3851 |
| VC2272 | *nrdR* | conserved hypothetical protein | 1.8579 |
| VC2323 |  | conserved hypothetical protein | -1.2272 |
| VC2357 |  | hypothetical protein | 1.3619 |
| VC2363 | *thrB* | homoserine kinase | 1.0090 |
| VC2364 | *thrA* | aspartokinase I/homoserine dehydrogenase threonine-sensitive | 1.3909 |
| VC2367 |  | hypothetical protein | -1.1229 |
| VC2373 | *gltD* | glutamate synthase large subunit | 1.1259 |
| VC2417 | *recJ* | single-stranded-DNA-specific exonuclease RecJ | -1.0980 |
| VC2418 | *dsbC* | thiol:disulfide interchange protein DsbC | -1.1996 |
| VC2419 | *xerD* | integrase/recombinase XerD | -1.1735 |
| VC2466 | *rseA* | sigma-E factor negative regulatory protein RseA | -1.1295 |
| VC2486 |  | hypothetical protein | -1.0348 |
| VC2490 | *leuA* | 2-isopropylmalate synthase | 1.1345 |
| VC2508 | *argF* | ornithine carbamoyltransferase | -1.4866 |
| VC2509 |  | hypothetical protein | -1.0323 |
| VC2510 | *pyrB1* | aspartate carbamoyltransferase catalytic subunit | 1.3190 |
| VC2511 | *pyrB2* | aspartate carbamoyltransferase regulatory subunit | 1.3940 |
| VC2524 | *ksdC* | conserved hypothetical protein | -1.1986 |
| VC2543 |  | hypothetical protein | 1.0762 |
| VC2544 | *fbp* | fructose-16-bisphosphatase | 1.6136 |
| VC2560 | *cysN* | sulfate adenylate transferase subunit 2 | 1.4625 |
| VC2562 | *cpdB* | 2`3`-cyclic-nucleotide 2`-phosphodiesterase | 1.2058 |
| VC2568 | *fklB* | peptidyl-prolyl cis-trans isomerase FKBP-type | 1.0420 |
| VC2637 |  | peroxiredoxin family protein/glutaredoxin | -1.3779 |
| VC2644 | *argC* | N-acetyl-gamma-glutamyl-phosphate reductase | -1.2890 |
| VC2645 | *argE* | acetylornithine deacetylase | -1.0803 |
| VC2656 | *frdA* | fumarate reductase flavoprotein subunit | 1.1030 |
| VC2657 | *frdB* | fumarate reductase iron-sulfur protein | 1.3599 |
| VC2658 | *frdC* | fumarate reductase 15 kDa hydrophobic protein | 1.7078 |
| VC2659 | *frdD* | fumarate reductase 13 kDa hydrophobic protein | 1.6990 |
| VC2674 | *hslU* | protease HslVU ATPase subunit HslU | -1.3301 |
| VC2675 | *hslV* | protease HslVU subunit HslV | -1.2577 |
| VC2699 | *dcuA* | C4-dicarboxylate transporter anaerobic | 1.0402 |
| VC2738 | *pckA* | phosphoenolpyruvate carboxykinase | 1.0864 |
| VCA0011 | *malT* | malT regulatory protein | 1.8819 |
| VCA0013 | *malP* | maltodextrin phosphorylase | 1.7128 |
| VCA0014 | *malQ* | 4-alpha-glucanotransferase | 1.6976 |
| VCA0015 |  | hypothetical protein | 1.6295 |
| VCA0016 |  | 14-alpha-glucan branching enzyme | 1.6418 |
| VCA0025 |  | transporter NadC family | 1.2441 |
| VCA0245 | *cmtB* | PTS system IIA component | 1.1053 |
| VCA0246 | *sgaT* | SgaT protein | 1.0727 |
| VCA0344 |  | hypothetical protein | 1.0116 |
| VCA0511 | *nrdG* | anaerobic ribonucleoside-triphosphate reductase | 1.1748 |
| VCA0516 | *ptsIIB* | PTS system fructose-specific IIBC component | 2.8382 |
| VCA0523 | *cqsA* | aminotransferase class II | 2.5848 |
| VCA0550 |  | hypothetical protein | -1.0960 |
| VCA0551 |  | hypothetical protein | -1.3942 |
| VCA0592 | *nudG* | MutT/nudix family protein | 1.6609 |
| VCA0628 | *secA* | SecA-related protein | 1.5356 |
| VCA0752 | *trx2* | thioredoxin 2 | -1.2519 |
| VCA0773 |  | methyl-accepting chemotaxis protein | 1.2086 |
| VCA0819 | *groES* | chaperonin 10 Kd subunit | -1.2272 |
| VCA0820 | *groEL* | chaperonin 60 Kd subunit | -1.1192 |
| VCA0821 |  | hypothetical protein | -1.1160 |
| VCA0823 | *ectC* | ectoine synthase | 1.3045 |
| VCA0824 | *ectB* | diaminobutyrate--pyruvate aminotransferase | 1.8200 |
| VCA0825 | *ectA* | L-24-diaminobutyric acid acetyltransferase | 1.6908 |
| VCA0867 | *ompW* | outer membrane protein OmpW | 1.6387 |
| VCA0897 | *devB* | devB protein | -1.1273 |
| VCA0912 | *exbD1* | TonB system transport protein ExbD1 | 2.0235 |
| VCA0944 | *malF* | maltose ABC transporter permease protein | 1.8529 |
| VCA0945 | *malE* | maltose ABC transporter periplasmic maltose-binding protein | 1.9859 |
| VCA0965 |  | GGDEF family protein | -1.3963 |
| VCA0966 |  | hypothetical protein | -1.3352 |
| VCA0985 |  | oxidoreductase/iron-sulfur cluster-binding protein | -1.3809 |
| VCA1006 |  | organic hydroperoxide resistance protein putative | -1.1304 |
| VCA1007 |  | hypothetical protein | -1.0642 |
| VCA1027 | *malM* | maltose operon periplasmic protein putative | 1.0597 |
| VCA1028 | *lamB* | maltoporin | 2.4851 |
| VCA1069 |  | methyl-accepting chemotaxis protein | 1.3835 |
| VCA1099 |  | ABC transporter permease protein | 1.0806 |

| **Table S4. Genes specifically affected by the *ribN* deletion.** | | | |
| --- | --- | --- | --- |
| **Gene**  **ID** | **Gene**  **Name** | **Gene Description** | **Fold Change (Log 2)** |
| VC0028 |  | dihydroxy-acid dehydratase | -1.0867 |
| VC0102 |  | hypothetical protein | -1.1603 |
| VC0366 | *rpsF* | ribosomal protein S6 | 1.1520 |
| VC0367 |  | primosomal replication protein N | 1.1356 |
| VC0368 | *rpsR* | ribosomal protein S18 | 1.0990 |
| VC0382 |  | hypothetical protein | 1.0603 |
| VC0383 |  | hypothetical protein | 1.1982 |
| VC0438 |  | conserved hypothetical protein | -1.1136 |
| VC0503 |  | conserved hypothetical protein | -1.6674 |
| VC0546 |  | hypothetical protein | -1.2149 |
| VC0625 |  | hypothetical protein | -1.1401 |
| VC0651 |  | conserved hypothetical protein | -1.7495 |
| VC0652 |  | protease putative | -2.0545 |
| VC0654 |  | conserved hypothetical protein | -1.4708 |
| VC0655 |  | acetyltransferase putative | -1.1821 |
| VC0706 |  | sigma-54 modulation protein putative | -1.2968 |
| VC0754 |  | conserved hypothetical protein | -1.0789 |
| VC0765 |  | conserved hypothetical protein | -1.4752 |
| VC0878 | *rpmE2* | ribosomal protein L31P family | -1.2763 |
| VC0879 | *rpmJ* | ribosomal protein L36 putative | -1.1210 |
| VC0895 |  | hypothetical protein | -1.1897 |
| VC1049 | *aphB* | transcriptional regulator LysR family | -1.1110 |
| VC1075 |  | conserved hypothetical protein | -1.0860 |
| VC1077 |  | hypothetical protein | -1.1365 |
| VC1114 | *bioC* | biotin synthesis protein BioC | -1.5557 |
| VC1115 | *bioD* | dethiobiotin synthetase | -1.7496 |
| VC1175 |  | hypothetical protein | 1.1525 |
| VC1226 |  | thiopurine methyltransferase | -1.3437 |
| VC1227 |  | hypothetical protein | -1.2496 |
| VC1248 |  | methyl-accepting chemotaxis protein | 1.3549 |
| VC1324 |  | hypothetical protein | 1.1045 |
| VC1343 |  | peptidase M20A family | -1.3345 |
| VC1563 |  | conserved hypothetical protein | 1.0680 |
| VC1564 |  | hypothetical protein | 1.1545 |
| VC1565 | *tolC* | outer membrane protein TolC putative | 1.2020 |
| VC1719 | *torR* | DNA-binding response regulator TorR | -1.7179 |
| VC1731 |  | conserved hypothetical protein | -1.0844 |
| VC1865 |  | hypothetical protein | -1.3762 |
| VC1871 |  | conserved hypothetical protein | -1.0342 |
| VC1950 |  | biotin sulfoxide reductase | -1.7854 |
| VC1951 | *yecK* | cytochrome c-type protein YecK | -1.8541 |
| VC1972 | *menA* | o-succinylbenzoate-CoA synthase | -1.5869 |
| VC1973 | *menB* | naphthoate synthase | -2.4451 |
| VC1974 | *menH* | conserved hypothetical protein | -2.1286 |
| VC2051 | *ccmG* | cytochrome c biogenesis protein | -1.1307 |
| VC2052 | *ccmF* | cytochrome c-type biogenesis protein CcmF | -1.3059 |
| VC2053 | *ccmE* | cytochrome c-type biogenesis protein CcmE | -1.8281 |
| VC2054 | *ccmD* | heme exporter protein D | -1.7078 |
| VC2055 | *ccmC* | heme exporter protein C | -1.4902 |
| VC2149 |  | hypothetical protein | -1.0069 |
| VC2361 | *grcA* | formate acetyl transferase-related protein | 1.0924 |
| VC2368 | *arcA* | aerobic respiration control protein FexA | -1.4087 |
| VC2371 |  | conserved hypothetical protein | -1.3033 |
| VC2372 |  | hypothetical protein | -1.3952 |
| VC2565 | *elaA* | elaA protein | -1.1079 |
| VC2689 | *pfkA* | 6-phosphofructokinase isozyme I | -1.0758 |
| VCA0053 | *ppnP* | purine nucleoside phosphorylase | 1.0621 |
| VCA0087 |  | hypothetical protein | -1.0036 |
| VCA0139 |  | hypothetical protein | -1.2355 |
| VCA0180 | *pepT* | peptidase T | -1.3636 |
| VCA0268 |  | methyl-accepting chemotaxis protein | 1.1518 |
| VCA0269 |  | decarboxylase group II | 1.2184 |
| VCA0621 |  | transcriptional regulator SorC family | -1.2832 |
| VCA0665 | *dcuC* | C4-dicarboxylate transporter anaerobic | -1.5123 |
| VCA0721 |  | hypothetical protein | -1.0138 |
| VCA0784 |  | hypothetical protein | -1.5655 |
| VCA0954 | *cheV* | chemotaxis protein CheV putative | -1.0292 |
| VCA0979 |  | methyl-accepting chemotaxis protein | 1.0063 |
| VCA0981 |  | hypothetical protein | 1.0077 |
| VCA1009 |  | hypothetical protein | -1.2601 |
| VCA1010 |  | conserved hypothetical protein | -3.4026 |
| VCA1014 |  | hypothetical protein | 1.0799 |
| VCA1063 | *speC* | ornithine decarboxylase inducible | 1.0672 |
| VCA1064 |  | hypothetical protein | 1.3655 |
